# Effect of Silicon on Dynamic/Static Corrosion Resistance of T91 in Lead–Bismuth Eutectic at 550 °C

**DOI:** 10.3390/ma15082862

**Published:** 2022-04-14

**Authors:** Ji Li, Xikou He, Bin Xu, Zhengxin Tang, Caishun Fang, Gang Yang

**Affiliations:** 1 Research Institute of Special Steel, Central Iron and Steel Research Institute, Beijing 100081, China; liji1988x@163.com (J.L.); tangzhengxin@nercast.com (Z.T.); yanggang@nercast.com (G.Y.); 2 Nuclear Power Institute of China, Chengdu 610014, China; xubilly@163.com (B.X.); sethfang0116@sina.com (C.F.)

**Keywords:** LBE, dynamic corrosion, silicon, static corrosion, LFR, 9Cr steel

## Abstract

The 9–12% Cr ferritic–martensitic heat-resistant steel is the main candidate structural material for the Lead-cooled Faster Reactor. The lower Gibbs free energy change of Si oxide can promote the formation of a stable oxide layer, which can improve the corrosion resistance of the material. Therefore, it is of great significance to study the effect of silicon (Si) on the corrosion resistance of T91 steel in lead–bismuth eutectic (LBE). The corrosion resistance of T91 steel with Si contents of 0.5 wt.%, 1.3 wt.%, and 2.0 wt.%, both in dynamic and static LBE at 550 °C, was investigated. The microstructure was analyzed by X-ray diffraction (XRD), scanning electron microscope (SEM) and transmission electron microscope (TEM), while the oxide films were characterized by electron probe microanalysis (EPMA). Results show that the addition of Si is conducive to improving the corrosion resistance of T91 steel in LBE. T91 steel with high Si content has a thinner and more stable oxide film. The change of Si content can change the contact angle between the steel and LBE, and the contact angle is the largest when the Si content is 1.3 wt.%. The Si-rich oxide layer is usually located in the inner oxide layer, which promotes the formation of a Cr oxide layer located in the internal oxidation zone (IOZ). Si will not enter the precipitated phase, but only change the ferrite content. The oxidation model of T91 steel containing Si in LBE was also proposed.

## 1. Introduction

As one of the most ideal candidate materials for the spallation target and coolant for accelerator driven subcritical system (ADS) [1,2,3,4,5,6], liquid lead–bismuth eutectic (LBE) provides a greater safety margin for the reactor due to its high boiling point and chemical inertness. However, the compatibility between LBE and structural materials has been a critical restriction in the lead-cooled faster reactor (LFR) development. The structural materials can be significantly influenced by the service condition, such as fast neutron irradiation, corrosion and erosion of high-temperature LBE and stress (thermal stress, processing stress, etc.). The LFR has the following performance requirements for its structural materials: first, good high-temperature mechanical properties, especially in the temperature range of 350–550 °C, since the cooling inlet/outlet temperature of the LFR is about 400/550 °C. Second, excellent LBE corrosion resistance because the LBE will cause the corrosive thickness on materials to reduce. Third, excellent resistance to liquid metal embrittlement because the synergistic action of LBE and stress will lead to material embrittlement.

To date, European and American researchers have carried out considerable screening and research and development on structural materials used in LBE medium. Commercial ferritic–martensitic heat-resistant steel, mainly represented by T91 steel, is the major candidate structural material for LFR [7,8,9,10], considering the comprehensive properties, industrial scale preparation, processability and application basis in the nuclear field.

Gibbs free energy is lower in the oxide film formed by Si and Al, which are the elements that can form a more stable oxide film than Cr. With the addition of Al, it is easy to form a large amount of ferrite, thereby damaging the mechanical properties of the material; for this reason, Al should be added carefully in F/M steel. In recent years, the corrosion behavior of 9–12% Cr F/M steel in LBE has been widely studied, especially the role of different alloy elements in the oxide film [11,12,13,14,15,16,17,18]. It is found that the addition of Si can appropriately improve the LBE corrosion resistance. Wang et al. [19] studied the resistance of 9Cr2WVTa steel with 0–1.36% Si to LBE at 550 °C, and found that the Si content had little effect on the macro-structure of the oxide film, which was mainly divided into three layers: the outer layer was PbO·xFe_2_O_3_; the secondary outer layer was FeCr_2_O_4_; and the inner layer was a mixture of Cr_2_O_3_ and SiO_2_. The dense inner film can hinder the diffusion of elements and the growth of an iron-rich outer oxide film, which improves the resistance to corrosion. Allen et al. [20] evaluated four ferroalloys with Si contents ranging from 0% to 3.82%, exposed in flowing LBE at 550 °C for 700–1000 h, and found three forms of Si: silicate at the top of the oxide layer; SiO_2_ close to the oxide-metal interface; and solid solution Si in the alloy. Alloys with low Si concentrations only contained silicate. Alloys with high Si concentrations contained a layer of SiO_2_ between oxide and metal, and several alloys with different Si contents showed oxidation failure.

Kurata Yuji et al. [21] studied the corrosion behavior of 316L and T91 steels containing 2.5% Si in LBE for 1200 h, and found that ordinary 316L and 316LSi steel could form oxide films to protect the matrix from LBE corrosion, and that the film of 316LSi is thinner at high oxygen concentrations (mass fraction of 2.5 × 10^−5^%). The two steels have a lead–bismuth permeation layer and a ferritic layer at low oxygen concentrations (mass fraction of 4.4 × 10^−8^%). The addition of Si significantly reduced the transformation from austenite to ferrite caused by nickel diffusion, whereas it could not completely prevent the LBE penetration. At low oxygen concentrations (mass fraction of 4.4 × 10^−8^%), however, the increase of Si content cannot effectively improve the LBE corrosion resistance of T91. Liu et al. [22] studied the corrosion resistance of SIMP and T91 steels in static LBE under 600 °C in two oxygen environments. Results revealed that SIMP and T91 steels in the low-oxygen environment formed thin oxide films, and that SIMP film was thinner. The oxide film of both steels in the saturated-oxygen environment is thicker, while that of SIMP is still thinner with a small amount of LBE infiltration.

There is no unified standard for LBE corrosion testing due to the wide experimental conditions. Thus, it is difficult to make any horizontal comparisons in the above research results. Meanwhile, there are few studies on the corrosion behavior of T91 steel with differing element content and various microstructures in LBE environment.

In this paper, three T91 steels with 0.5% Si, 1.3% Si and 2.0% Si, named T91LSi, T91MSi and T91HSi, respectively, were exposed to flow LBE for 1500 h and to static LBE for 50–1000 h. The microstructures of virgin materials were analyzed with the use of an optical microscope and SEM, while the thickness and composition distribution of oxide film formed in LBE were characterized. The Si effect on the oxide film of T91 steel was studied and the influence mechanism was discussed to obtain the influence law of Si on the LBE corrosion resistance of T91 steel, which could provide technical support for the design and structural material selection of LFR.

## 2. Materials and Methods

### 2.1. Materials

T91LSi, T91MSi and T91HSi with an ingot size of 120 mm, were produced by a 25 kg vacuum induction furnace. In order to control the chemical composition accurately, the smelting process needs to be sampled and adjusted in real time. The three steels were heated to 1100 °C for 2 h, then forged at final temperature of over 900 °C into 13 mm plates, followed by annealing, and then normalizing at 1050 °C for 0.5 h, followed by tempering at 770 °C for 2 h according to ASME SA213-2019. The chemical composition of the steels was analyzed by chemical titration according to GB/T 223-2006 and the result is given in Table 1.

### 2.2. Thermodynamic Calculation

Thermodynamic calculation was carried out to study the effect of Si on the microstructure of T91 steel. Firstly, the equilibrium phase diagram of T91 steel with Si content in the range of 0–2.5% was calculated at temperatures of 400–1600 °C. The temperature range was divided into 100 steps equally. TC-Fe11 material database was selected to provide support for microstructure analysis. The calculated system was set at standard atmospheric pressure with the total amount of 1 mol. Then, the same system and steps were used to calculate the changes of each phase of T91 with silicon content of 0.48%, 1.30% and 1.95% at different temperatures.

### 2.3. Structure Observation

The heat-treated plates were obtained by wire cutting, ground to a roughness of 106 μm, 45 μm, 23 μm and 13 μm, and then polished, followed by soaking in aqueous solution of CuCl_2_ and hydrochloric acid for 10 s. The original structure of the steels were observed by a digital metallographic microscope (Zeiss 40MAT, Carl Zeiss AG, Oberkochen, Germany), at 50 times of magnification.

A thermal field emission scanning electron microscope (FEI-Quanta650, Thermo Fisher Scientific, Waltham, MA, USA) was used to observe the above samples at a magnification of over 1000 times. The SEM mode was secondary electronic imaging, with the working distance 10–15 mm, the magnification 5000 times, and the acceleration voltage 20 kV.

The microstructure was characterized by transmission electron microscope (TEM, Tecnai G2 F20 S-TWIN, Thermo Fisher Scientific, Hillsboro, OR, USA). The steel block samples were cut to 0.5 mm, then thinned by 13 μm sandpaper to 50 μm, then cut into a circle with a diameter of 3 mm. The Automatic Twin-Jet Electropolisher (MTP-1A, BUEHLER, Lake bluff, IL, USA) was used for double spray, with the current 40 mA, the voltage 20 V, the experimental temperature −20 °C, and the experimental solution 10% perchloric acid. The obtained samples were observed at an operating voltage of 200 kV.

### 2.4. Phase Analysis

The steels were electrolyzed and the precipitated phases extracted, which were analyzed by XRD measurement. The carbides were extracted with ammonium sulfate and citric acid aqueous solution with a current density of 0.03 A/cm^2^ at 15–20 °C. The intermetallic phases were extracted with HCl + glycerol + citric acid methanol solution with a current density of 0.05 A/cm^2^ at a temperature between zero to minus 5 °C.

The washed and dried electrolytic residue was taken as the sample and analyzed by X-ray diffractometer with the diffraction conditions as follows: Panaco X Pert MPD diffractometer, 2*θ* angle 20–120°; anti-scattering slit 5.5 mm; step size 0.0167°, time 20 s; array detector; Cu target; pipe voltage 40 kV and pipe electric current 40 mA.

Above methods were adopted to determine the diffraction d value and relative intensity of electrolytic residue. Jade software application was used to analyze the XRD results. Then the precipitated phases were analyzed.

Strong acid was used to dissolve the residue of the precipitated phase after electrolysis and then the liquid sample was obtained. The mass fraction of each element in the precipitate was measured quantitatively by inductively coupled plasma emission spectrometer (ICP), and then converted into atomic fraction.

### 2.5. Corrosion Test

Three 30 × 10 × 2 mm^3^ sheet samples were exposed in the dynamic LBE corrosion circuit at 550 °C with the oxygen content of 10^−6^–10^−7^ wt.% at flow rate of 2–5 cm/s for 1500 h. Fifteen 30 × 10 × 2 mm^3^ sheet samples were corroded in the static 550 °C LBE kettle with saturated oxygen for 50 h, 100 h, 200 h, 500 h and 1000 h.

### 2.6. Oxide Film Characterization

Steels corroded by LBE were analyzed by electron microprobe, and then the structure and composition distribution of oxide films were characterized. Shimadzu EPMA-1720H was used for analysis with the X-ray spectrometer 2–5 channels, the X-ray extraction angle 52.5°, the radius of Roland circle 101.6 mm, and the secondary electron resolution 5 nm.

### 2.7. LBE and Wettability Test

**Figure 1:**
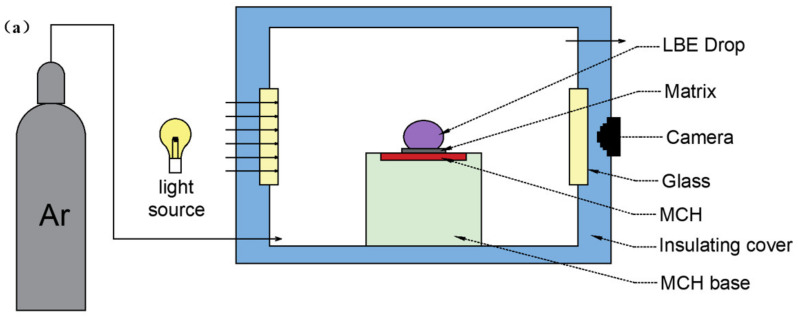

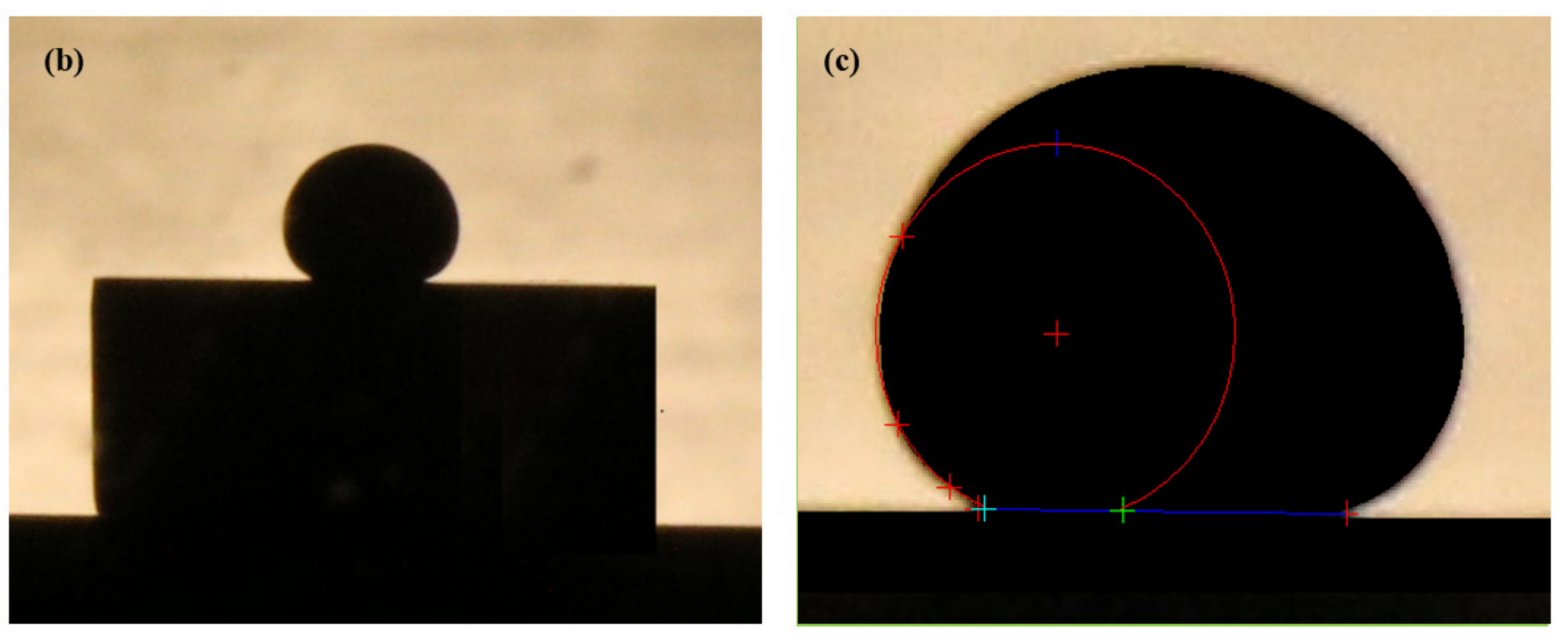
(**a**) Measurement of contact angle. (**b**) Side view. (**c**) Five-point fitting measurement image.

The contact angle between solid and liquid was measured to evaluate the wettability between LBE and stainless steels. The experimental device is shown in Figure 1a. High-purity argon was filled into the sealed box as the protective gas before the test. Supplemental light was added from the back and the heating plate was installed directly below the sample substrate. The droplets were deposited on the sample, and the image shown in Figure 1b was obtained from the side view. Image processing software JC2000DB and five-point fitting method were adopted to process the droplet image to obtain the static contact angle [23], as shown in Figure 1c.

## 3. Results

### 3.1. Thermodynamic Calculation

**Figure 2:**
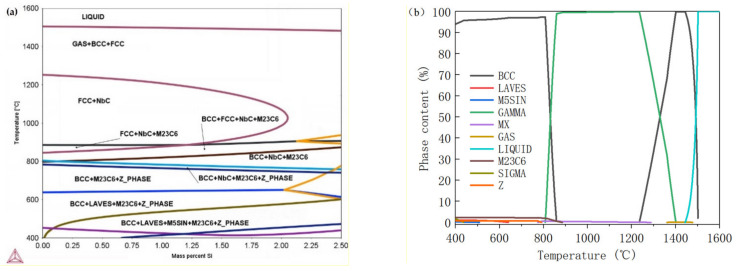

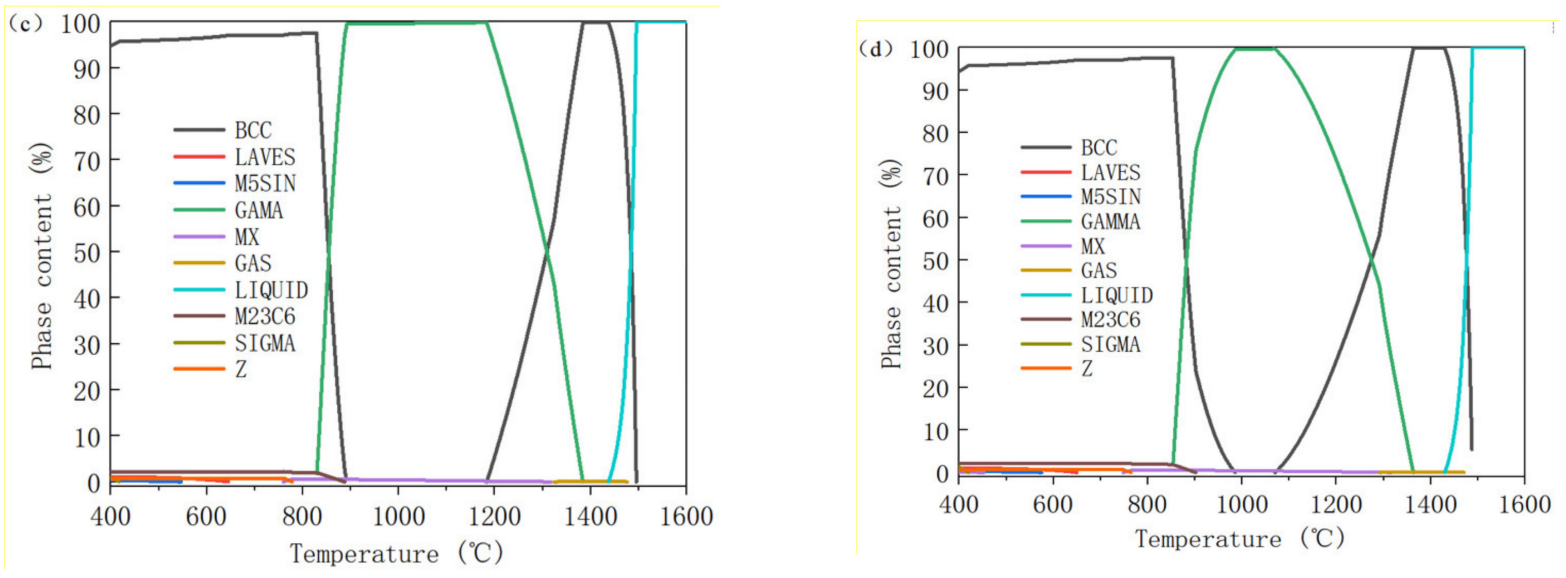
Thermal-Cal results. (**a**) Equilibrium phase diagram. (**b**–**d**) Precipitate change of T91LSi, T91MSi, and T91HSi with temperature.

T91 steel (Figure 2) is mainly austenite at high temperature, and martensite after cooling with precipitated M_23_C_6_ and MX phases. The content of M_23_C_6_ phase, which is the carbide of Fe, Cr and Mo, is higher than the MX phase with mainly component of Nb (C_x_N_1-x_). The content change of Si has little impact on the type, but does have a little impact on the proportion of precipitates when the content of Si is less than 2.0 wt.%. M_23_C_6_ will transform to M_6_C phase with significantly increased ferrite content when the content of Si exceeds 2.0 wt.%. In the equilibrium phase diagram (Figure 2a), Z phase and Laves phase are precipitated after long-term aging, but the intermetallic phases are not precipitated due to an insufficient kinetic condition in 1000 h. [24,25] The effect of a Si increase on the material structure is mainly reflected in an increase in ferrite ratio, that is, the shrinkage of the complete austenitization temperature range has little impact on carbide and the intermetallic phase, as shown in Figure 2b–d. Therefore, the optimum normalizing temperature for each Si content is 1050 °C.

### 3.2. Structure Observation

**Figure 3:**
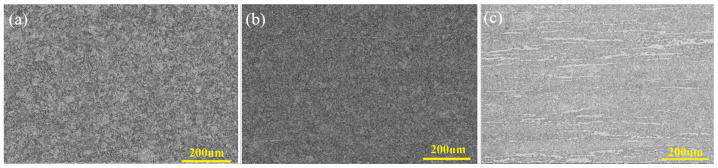

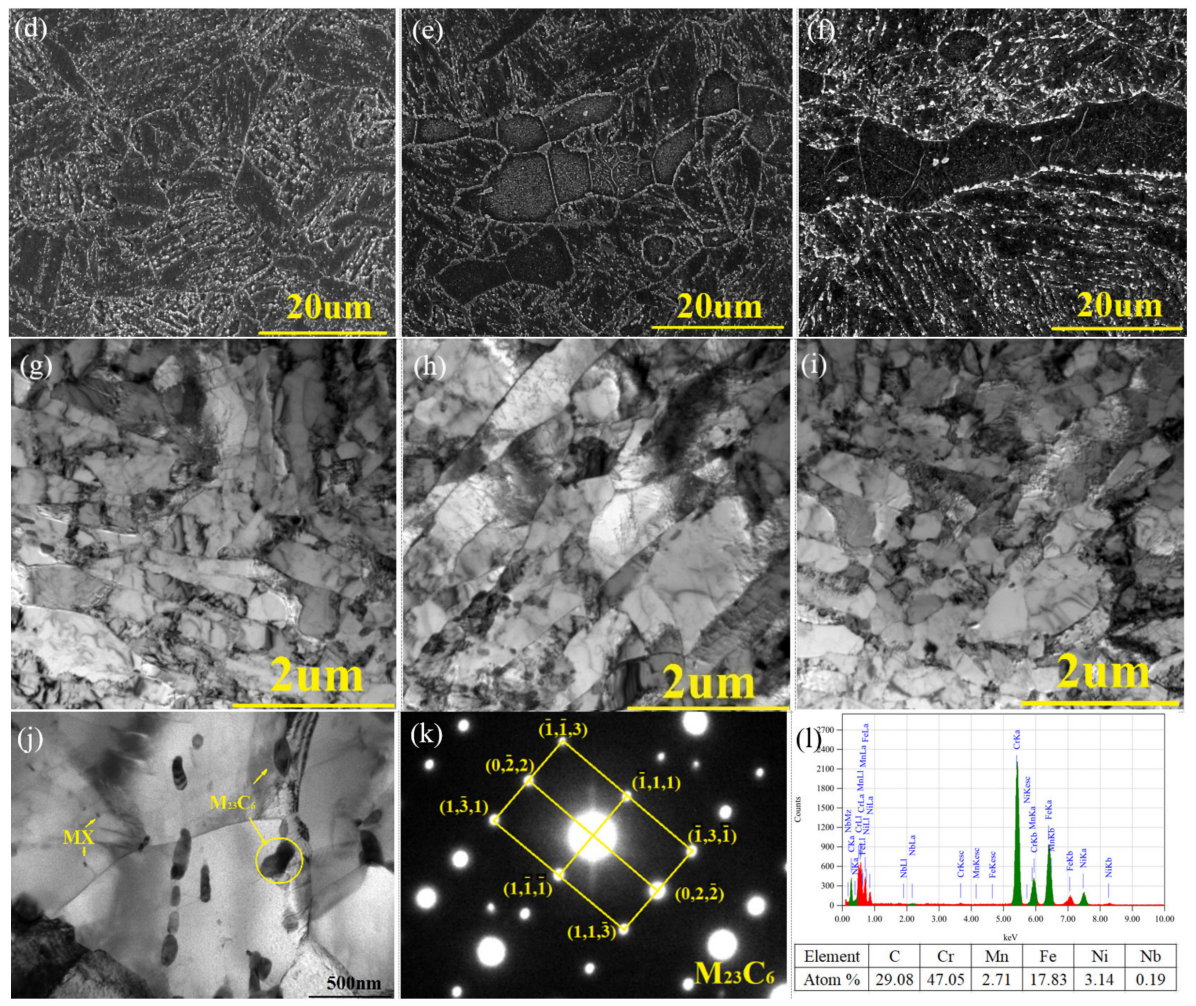
Microstructure of three steels: (**a**) T91LSi OM; (**b**) T91MSi OM; (**c**) T91HSi OM; (**d**) T91LSi SEM; (**e**) T91MSi SEM; (**f**) T91HSi SEM; (**g**) T91LSi TEM × 20,000; (**h**) T91MSi TEM × 20,000; (**i**) T91HSi TEM × 20,000; (**j**) T91MSi TEM × 50,000; (**k**) SAED result for mark points in image “**j**”; (**l**) EDS result for mark points in image “**j**”.

Figure 3a–c show that T91LSi, T91MSi, T91HSi steels have a tempered martensite structure with a fine grain size, grade 8.0. T91MSi and T91HSi steels contain banded ferrite with the content of about 1% and 12%, respectively. As shown in Figure 3d, the carbides of T91LSi steel are mainly distributed at the prior austenite grain boundary and lath boundary. Figure 3e,f show that a large number of carbides are distributed around the ferrite of T91MSi and T91HSi steels, and a small quantity of carbides precipitate in the ferrite, which will have a certain impact on the mechanical properties and corrosion resistance.

As shown in Figure 3g–i, M_23_C_6_ and MX carbides mainly exist at the prior austenite grain boundary, the packet boundary, the block boundary and the lath boundary. The size of M_23_C_6_ is large, about 200 nm, while the size of MX is very small, only 20 nm. When the content of N/C atoms and V/Nb atoms in the matrix exceeds its solubility product, supersaturated alloy elements will form the MX phase, which usually exists in the early stage of solid phase formation The formation temperature of MX in the T91 system is very high, about 1300 °C. MX phase is generally small, and is too stable in an aging process of tens of thousands of hours to grow. MX phase is a beneficial second phase to improve the mechanical properties of T91 steel at high temperatures over a long time period. When the temperature is lower than 900 °C, M_23_C_6_ phase will appear in the microstructure. M_23_C_6_ phase is relatively large, and has a significant pinning effect on grain boundaries, effectively hindering the movement of dislocations, which significantly improves the strength of the material. T91 is in service in a stress environment at high temperature; therefore, the stability of the precipitated phase plays a decisive role in its long-term service. The precipitated phases of the three steels are similar. In order to avoid tedious repetition, only the precipitated phase of T91MSi steel is shown in Figure 3j–l.

### 3.3. Phase Analysis

**Figure 4:**
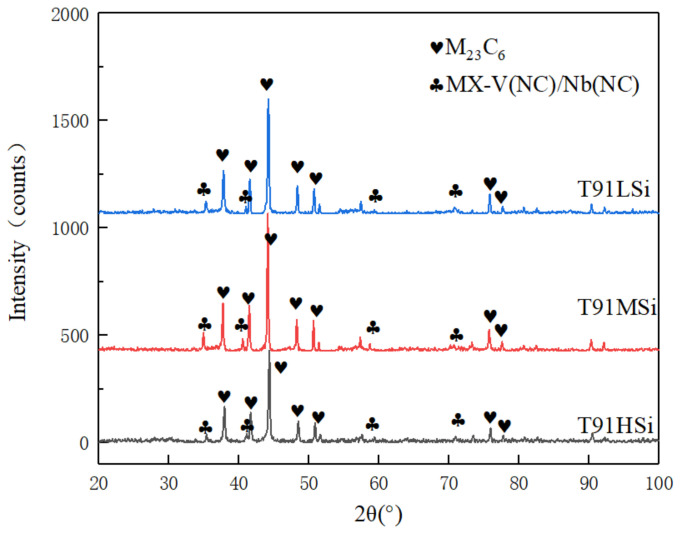
XRD measurement results of precipitates.

The phases analysis results of three steels are given in Table 2. The composition of ferrite and matrix is close to that of the matrix, which is decomposed in the electrolysis process, leaving only the second phase with a high content. The three steels have relatively similar types of precipitated phases: M_23_C_6_ phase and MX phase. The element proportion of M_23_C_6_ phase and MX phase are shown in Table 3 and Table 4, respectively. It can be seen that the M_23_C_6_ phase is the carbide of Fe, Cr, Mo, V, Mn and Ni, and the MX phase is the carbon nitride of V, Nb and Mo. The XRD measurement results of the steels after extraction are shown in Figure 4. The MX phase in the microstructure is easy to be affected by experimental errors due to its low content.

Based on the results of the Thermo-calc simulation, microstructure observation, and phase analysis, it is concluded that the change of 0–2 wt.% Si content has little effect on the precipitated phase of T91. The greatest effect of the increase of Si on the microstructure is reflected in the increase in ferrite content and the change in carbide distribution. The change of Si has little influence on the total amount of precipitated phase or the type of precipitated phase, but slightly affects the composition of the precipitated phase.

### 3.4. Characterization of Static Corrosion Oxide Film and Corrosion Kinetics

**Figure 5:**
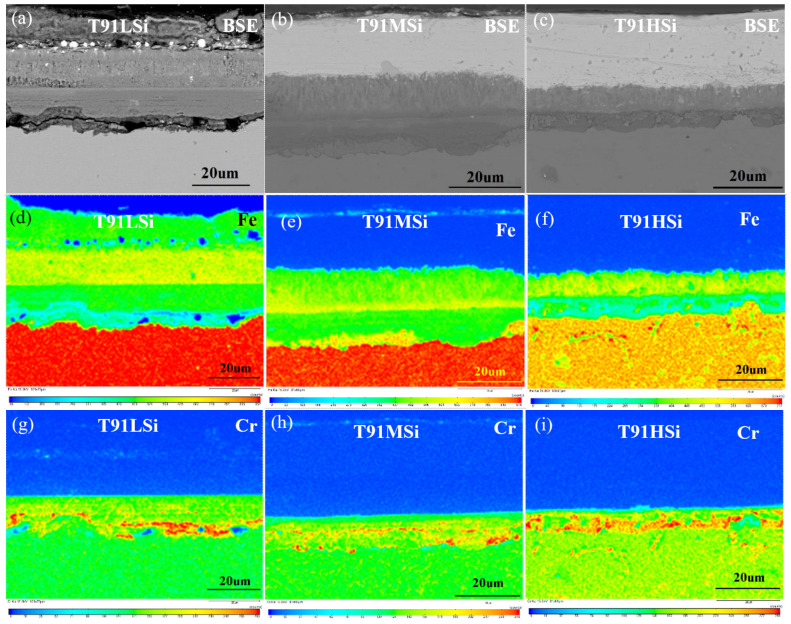

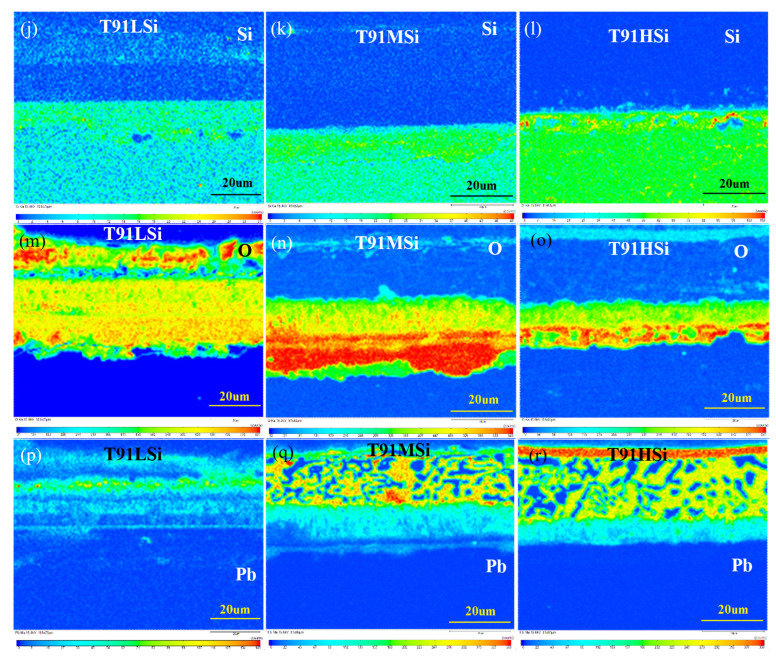
EPMA images of the oxide films for the three steels. (**a**,**d**,**g**,**j**,**m**,**p**) for T91LSi, (**b**,**e**,**h**,**k**,**n**,**q**) for T91MSi and (**c**,**f**,**e**,**l**,**o**,**r**) for T91HSi. (**a**–**c**) BSE photos. (**d**–**f**) Distribution of Fe elements. (**g**–**i**) Distribution of Cr element. (**j**–**l**) Distribution of Si element. (**m**–**o**) Distribution of O element. (**p**–**r**) Distribution of Pb elements. The color from cold to warm indicates a component content from low to high.

EPMA analysis was carried out on three steels after corrosion for 50 h, 100 h, 500 h, and 1000 h. The result for 1000 h results are shown in Figure 5. It can be seen that the surfaces of the three steels all show oxidation and infiltration corrosion. The backscatter photos (Figure 5a–c) show that the T91LSi steel has the thickest oxidation layer, 42 μm, while T91MSi 22 μm and T91HSi the thinnest, only 15 μm.

Based on the morphology analysis, the oxide film of T91LSi is relatively loose, with a large number of diffusion channels. The matrix atoms and lead and bismuth atoms can pass through the oxide film smoothly, resulting in further corrosion. The oxide film of T91MSi has good continuity and a stable overall structure. The oxide film of T91HSi also has good continuity, but the Fe, Cr and Si elements are enriched at the grain boundary.

Based on the composition analysis, the composition distribution law of oxide films of the steels with different Si content is the same, but the composition of each layer is very different. The oxide film is divided into an outer layer and an inner layer, according to its relationship with the original matrix surface. The oxide layer growing from the original surface to the LBE side is called the outer layer, which is divided into an outermost layer and a sub-outer layer. The oxide layer corroded and infiltrated from the original surface to one side of the substrate forms the layer below the original surface, which is temporarily divided into an inner layer and an internal oxidation zone (IOZ).

The T91LSi oxide film is distributed in four layers, as shown in Figure 5a,d,g,j,m,p. The outermost layer with thickness of 15 μm is mainly Fe and a small amount of Si, with a large amount of lead–bismuth infiltrated. The secondary layer with a thickness of 15 μm is mainly Fe, doped with a small amount of lead–bismuth. The content of Fe in the inner layer with a thickness of 8 μm decreases slightly, and the contents of Cr and Si increase slightly. The contents of Cr and Si in the IOZ reach the peak, and the Fe content is further reduced compared to the inner layer. The IOZ with thickness of 5 μm is mainly the composite of SiO_2_ and (Fe,Cr)_x_O_y_, with infiltrated Pb atoms.

The boundary between the outer layer and the sub-outer layer of T91MSi is not clear, as shown in Figure 5b,e,h,k,n,q. The thin outer layer with thickness 13 μm is mainly composed of Fe and Pb oxides, without Cr and Si infiltration. The inner layer is only 3 μm thick, and is mainly composed of Fe, Cr and Si oxides, with a little Pb. The IOZ is enriched by Cr and Si oxides and depleted by Fe oxides, without the infiltration of Pb. This layer is an important guarantee for the steel’s resistance to corrosion, and almost isolates the diffusion of Pb, Bi, Fe, Cr, and O. The thickness of IOZ in different position is 0–3 μm.

The oxide film of T91HSi is only a two-layer structure, as shown in Figure 5c,f,i,l,o,r. The outer lay with thickness of 7–8 μm is the oxide of Fe and Pb, and has high Fe content and low Pb content near the surface. The inner lay with thickness of 7–8 μm is thin mixed oxide of Fe, Cr and Si. Because there are many ferrites in the structure, a great deal of carbides are distributed at the boundary between ferrite and martensite, and there are more carbides at the original austenite grain boundary, which coarsens the grain boundary. At the same time, due to the smaller diffusion activation energy at the grain boundary, Fe atoms preferentially diffuse outward along the grain boundary where the Fe content is low.

Tian et al. [26] carried out corrosion tests on T91 steel at 550 °C LBR with oxygen content of 0.01 ppm, and found that the corrosion depth reached 59 μm. In this experiment, the corrosion depth is generally less than 59 μm due to the adjustment of Si content in T91.

The oxide film thickness of the three steels exposed in 550 °C LBE for different time was analyzed, and the average thickness is shown in Table 5.

**Figure 6:**
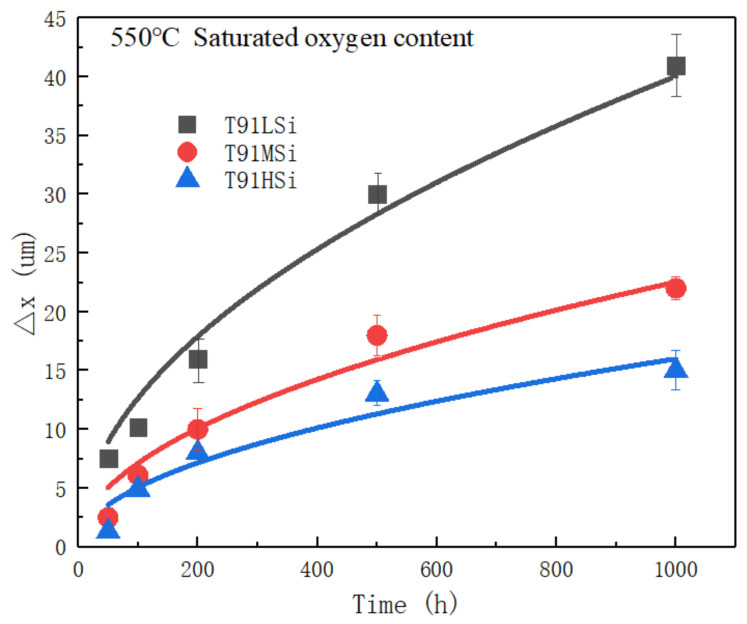
Variation law of oxide film thickness with time static LBE.

The corrosion kinetics curve is drawn for the variation law of oxide film thickness with time, as shown in Figure 6.

Fitting of data (Figure 6) based on Formula (1):

(1) $$\Delta X^{2}=k_{p}t$$

where, Δ*X* is the thickness of oxide film, *t* is the corrosion time (h); *k_p_* is the parabolic rate constant (μm^2^/h). With the increase of corrosion time, the thickness of oxide film on the surface of the three steels increases, but the increase rate decreases gradually with time. The parabolic rate constants *k_p_* of the three are 1.1253 μm^2^/h, 0.8442 μm^2^/h, 0.7114 μm^2^/h, respectively.

The smaller the *k_p_* value, the better the LBE corrosion resistance. It is concluded that the increase of Si reduces the *k_p_* value, which is conducive to the formation of stable oxide film and improves the LBE corrosion resistance.

### 3.5. Characterization of Dynamic Corrosion Oxide Film

**Figure 7:**
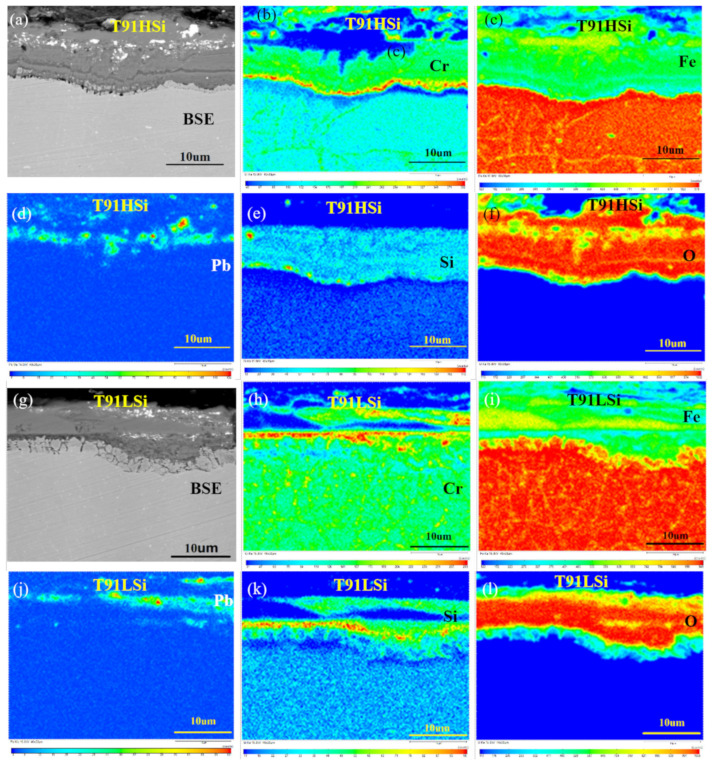
EPMA characterization of dynamic corrosion oxide film. (**a**–**f**) Composition distribution of T91HSi steel oxide film; (**g**–**l**) Composition distribution of T91LSi steel oxide film. The color from cold to warm indicates the component content from low to high.

After being exposed in the dynamic LBE circuit, the oxide films of steels show obvious erosion phenomenon, and the LBE further corrodes the deep structure after the outer oxide film falls off. Figure 7b–f shows that the outer layer of the oxide film of T91HSi steel is composed of Fe oxide, the inner part is mainly SiO_2_, and the IOZ is Cr_2_O_3_. Figure 7h–l show the outer layer of T91LSi steel is similar to that of T91HSi steel, but the inner and IOZ are disorderly distributed. Figure 7b,h show that T91HSi steel has better continuity of oxide film than T91LSi steel, and a better continuity of the intermediate Si-rich layer, but the Cr-rich layer is thinly distributed in the inner layer. Figure 7e,k show that T91HSi steel has a more uniform Si-rich layer than T91LSi steel. In the matrix near the IOZ, T91HSi steel has a continuous and clear Cr-rich layer due to the continuous distribution of carbides at the grain boundaries and a large amount of ferrite in the structure, while T91LSi steel has a relatively discrete distribution of the Cr-rich layer.

### 3.6. Wettability Test

**Figure 8:**
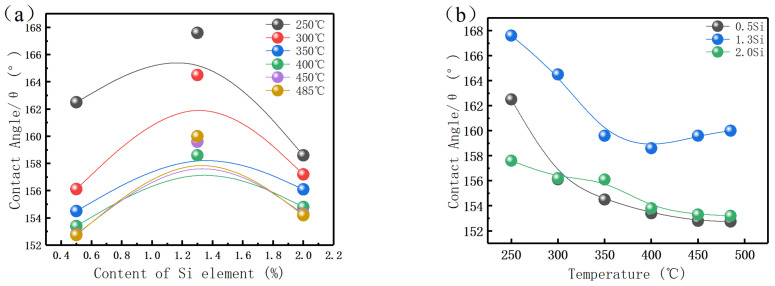
(**a**) Si effect on contact angle. (**b**) Temperature effect on contact angle.

It is believed that the corrosion resistance of the material and the liquid metal embrittlement (LME) phenomenon of ferritic–martensitic steel in LBE have a certain relationship with the wettability [27]. To explore the relationship between wettability and corrosion resistance, a contact angle measuring device is used to test the contact angle between the steel and LBE droplets at a temperature of 250–500 °C. The results are shown in Figure 8.

The contact angles (Figure 8a) decrease gradually with the increase in temperature, which is consistent with the greater corrosion tendency for higher temperatures. The decrease rate of the contact angle is larger in the range 250–400 °C, and smaller above 400 °C. Notably, when the Si content is 1.3%, the contact angle increases instead.

The contact angles (Figure 8b) increase with the increase in Si content, but decrease when the Si content exceeds 1.3%. This is because the addition of Si causes phase change in the structure.

**Figure 9:**
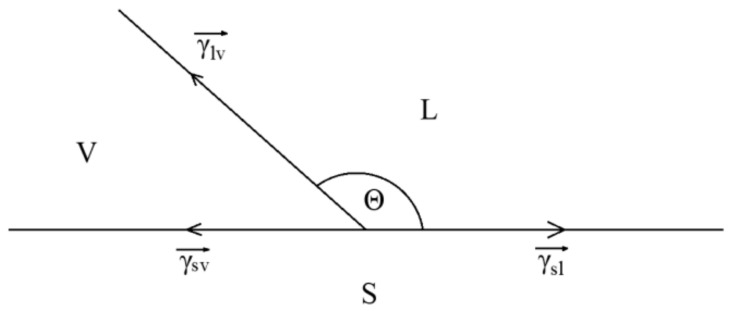
Relationship between contact angle and surface tension.

The relationship between contact angle and surface tension is shown in Figure 9. When the LBE droplet contacts the surface of steels, surface tension will be generated between the three interfaces, which are solid–liquid interfacial tension, gas–liquid interface tension, and solid–gas interface tension $\vec{\gamma_{sv}}$, $\vec{\gamma_{sl}}$, $\vec{\gamma_{vl}}$.

$\vec{\gamma_{vl}}$ of the three steels is the same, and $\vec{\gamma_{sv}}$ of the three steels is similar, but $\vec{\gamma_{sl}}$ is different because the change of surface composition after adding Si leads to the change in reaction products between LBE droplets and samples. When the structure is full martensite, the oxide film after corrosion becomes thinner due to the increase in Si content, and the oxide composition on the surface changes, which jointly affects the $\vec{\gamma_{sv}}$. When the microstructure contains a large amount of ferrite, the oxidation products change and the corroded crystal surface also changes, which has a great influence on the tension of the solid–liquid interface.

## 4. Discussion

The corrosion failure modes of materials in LBE are oxidation and dissolution, and the root cause is diffusion. On the one hand, the matrix material diffuses into LBE, on the other hand, oxygen diffuses into the matrix. The corrosion mechanism and oxide film behavior of 9–12% Cr steel in LBR were studied, and a more classical oxide film growth model was put forward [28]. In this study, the change of oxide film after the addition of Si is studied, the formation sequence of oxide film in different layers is analyzed from the perspective of the formation energy of various oxides, and a new model is proposed.

Excessively high oxygen content in LBE will produce a thick oxide film which can protect the material from further corrosion, but the falling off of the oxide film is easy to block the flow channel, thus affecting the reactor operation. It is hard to form a continuous oxide film in the oxygen-depleted LBE solution, so that the matrix cannot be protected from corrosion. Therefore, the ideal oxygen content in the alloy is of great significance for improving the corrosion resistance of structural materials in LBE.

Two oxygen-controlled environments were prepared to study the influence of Si content on oxide film: the dynamic LBE circuit with 10^−6^–10^−7^ wt.% oxygen, and the static LBE corrosion medium with saturated oxygen. The oxygen content is calculated as in Formula (2):

(2) $$logC_{o,max}\left(\mathrm{wt}.\%\right)\;=\;2.99-\frac{4711}{T}$$

The oxygen content in the oxygen-saturated LBE is 1.8 × 10^−3^ wt.% when the temperature is 823 K. Such a high oxygen content makes the LBE solution oxidize quickly after contacting the matrix, as shown in Formula (3):

3Fe(s) + 2O_2_(g) = Fe_3_O_4_(s) (3)

In contrast, a higher chemical potential of oxygen in oxygen-saturated LBE is conducive to the oxidation reaction which takes place on the surface of the material. In the reaction process, Fe ions continue to diffuse outward, and therefore a thicker Fe_3_O_4_ oxide film is formed with corrosion time. This film is porous, which cannot prevent the diffusion of Pb, Bi and O from outside to inside, nor can it prevent the further outward diffusion of Fe ions.

The oxygen will penetrate the sparse outer film and reach the vicinity of the original matrix surface, then combine with Cr in the matrix to form Cr oxide as follows:

4Cr(s) + 3O_2_(g) = 2Cr_2_O_3_(s)
Δ*G^θ^* = −773,173 + 148.5*T* (4)

It is calculated that when the temperature is 823 K, the Gibbs free energy (Δ*G^θ^*) is −650.96 kJ/mol. It can be seen that Cr can easily and spontaneously form Cr oxide after encountering O_2_. Cr oxide is usually distributed in flake shape, and its density is larger than Fe_3_O_4_, which has a certain protective effect on the matrix.

When the chemical potential of Fe ions and O ions near the oxide film is high enough, the unstable oxide film Cr_2_O_3_ will further react with Fe atoms as follows:

4Fe(s) + O_2_(g) + 2Cr_2_O_3_(s) = 2FeCr_2_O_4_(s) Δ*G^θ^* = −550,858 + 101.3*T* (5)

Δ*G^θ^* of this reaction is −467.49 kJ/mol. Thus, when oxygen diffuses near the surface of the oxide film, Cr will preferentially combine with oxygen to form Cr_2_O_3_, which is not continuous initially, and many positions cannot effectively block the diffusion of ions. After further reaction of Cr_2_O_3_ with surrounding Fe and O_2_, the spinel oxide FeCr_2_O_4_ is continuously lamellar, which effectively hinders the further diffusion of anions and cations.

When the matrix contains more Si, the oxygen in LBE will react with Si in the matrix after diffusing near the surface of the matrix through the outer layer as follows:

Si(s) + O_2_(g) = SiO_2_(s) Δ*G^θ^* = −907,100 + 157*T* (6)

Δ*G^θ^* of this reaction is −777.89 kJ/mol. It can be seen from the comparison of Gibbs free energy, calculated by Formula (4), that the oxidation reaction of Si is easier to spontaneously proceed than that of Cr. On the one hand, the formation of SiO_2_ and Cr_2_O_3_ on the original surface can hinder the outward diffusion rate of Fe atoms; on the other hand, it can also reduce the oxygen partial pressure at the original surface, so as to prevent further oxidation reaction of Fe, which is also one of the reasons for the thinning of oxide film when Si is increased. In general, the Si in the inner oxide layer is distributed near the surface, not near the matrix. Atkinson et al. [29] believe that Si oxide has a certain associated relationship with Cr oxide, because the SiO_2_ particles formed become the Cr_2_O_3_ nucleation, which is conducive to the Cr_2_O_3_ growth. Therefore, according to the EPMA (Figure 5 and Figure 7) results of corrosion test, the Cr-rich layer is often the innermost layer, and the Si-rich layer above it often has a high Cr content. Usually, SiO_2_ initially exists in the oxidation process in granular form, so it is difficult to form a completely protective oxide film in the initial stage or when the Si content is low. Its function is mainly to block the Fe_3_O_4_ channel, which is conducive to the formation of a stable FeCr_2_O_4_ spinel oxide film inside [29,30]. When the chemical potential of Fe and O_2_ near SiO_2_ oxide reaches the reaction conditions, the following reactions will occur further:

2Fe(s) + O_2_(g) + SiO_2_(s) = Fe_2_SiO_4_(s) Δ*G^θ^* = −566,095 + 143.9*T* (7)

Δ*G^θ^* of the reaction is −447.67 kJ/mol. Compared with the Gibbs free energy from Formula (5), the stability of SiO_2_ is slightly stronger than that of Cr_2_O_3_.

**Figure 10:**
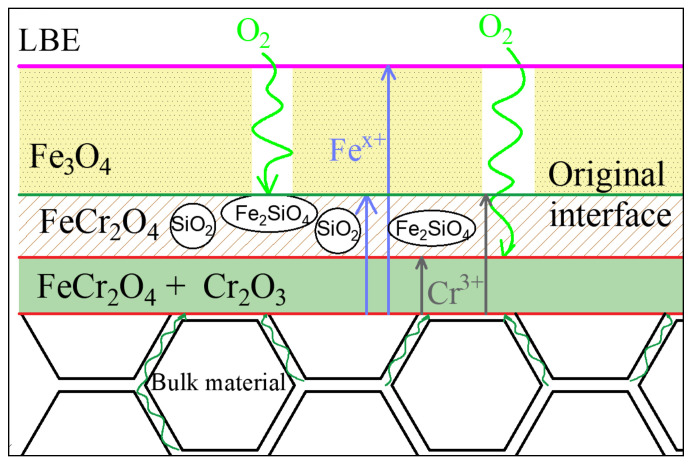
Oxidation of T91 containing Si.

In conclusion, T91 steel with different Si content has a similar oxide film structure after being oxidized in LBE. The oxide film is divided into three layers. The outer Fe_3_O_4_ layer is formed first due to the diffusion of Fe and O, which is the channel for LBE and O to corrode the matrix material inward. The inner SiO_2_ layer has the best stability of particles, and the number of particles is enough to create a continuous layer that can form a dense oxide film with FeCr_2_O_4_, which effectively hinders the diffusion of various ions. Fe_2_SiO_4_ formed after a few SiO_2_ reactions can reduce the chemical potential of Fe and O. In the IOZ, due to the addition of Cr, it forms FeCr_2_O_4_ spinel with Fe, which is continuous and dense. Due to the low chemical potential of Fe and O, the remaining Cr still exists as Cr_2_O_3_, which has become the innermost barrier to protect the matrix material. Therefore, the oxidation diffusion model is obtained, as shown in Figure 10.

When the Si content is high, the chemical potential on the left of the reaction Formula (6) increases, and the content of Si oxide in the oxide film of the inner layer also increases, which is conducive to the formation of oxide film. At the same time, the increase in Si leads to an increase in *δ* ferrite in the structure, and an increase in diffusion channels caused by the coarsening of grain and phase boundaries, which reduces the activation energy of Si, Cr, Fe and other types of diffusion at the surface. T91 steel *δ* ferrite is known to be harmful to mechanical properties, and the composition segregation degree and microstructure uniformity in the engineering process are quite different from those in the laboratory. Therefore, the Si element should be added according to the specific design materials.

The changes in oxide film, matrix and precipitated phase in the corrosion test for more than 2000 h need to be further discussed in subsequent research work, and the tests are under way.

## 5. Conclusions

1. The addition of Si effectively reduces the thickness of the oxide film. The thickness of oxide film decreases from 41 μm to 15 μm after exposure at 550 °C LBE for 1000 h. The increase in Si content can help improve the corrosion resistance of T91 steel in LBE. The addition of Si has little effect on carbide, and *δ* Ferrite will form when Si increases to 2.0 wt.%.
2. Corrosion resistance mainly depends on the three-layer film. The outermost film plays a lesser role, while the two films below the original surface play a major role, improving corrosion resistance by forming a stable and dense oxide that hinders diffusion channels. The outer layer Fe_3_O_4_ is porous. The intermediate composite layer of Si and Cr plays a critical role in stabilizing the oxide film, which also provides favorable conditions for the formation of Fe Cr spinel in IOZ.
3. The increase in Si content can increase the contact angle between the material and LBE droplets when the matrix structure remains unchanged. When ferrite exists in the structure, the dual phase structure of ferrite and martensite affects the interfacial tension between solid and liquid and reduces the contact angle.
4. The oxidation model of T91 steel containing Si in the LBE environment is obtained through the analysis of the oxidation process. The model reveals the path of atomic diffusion and the direction of oxide film growth, and shows the hindering effect of Fe_2_SiO_4_ on oxygen diffusion channel.

## Figures and Tables

**Table 1:** Chemical composition of test steels (wt.%).

Material	C	Si	Mn	P	S	Ni	Cr	Mo	V	Nb	N	Fe
T91LSi	0.10	0.48	0.49	0.004	0.001	0.35	9.05	0.88	0.19	0.08	0.059	Balance
T91MSi	0.10	1.30	0.47	0.004	0.001	0.37	8.97	0.89	0.20	0.08	0.060	Balance
T91HSi	0.10	1.95	0.47	0.004	0.001	0.36	9.03	0.88	0.18	0.08	0.058	Balance

Measurement errors: S and P ± 0.002%; N ± 0.004%; C, Mn, V, Nb ± 0.01%; Si and Ni ± 0.02%; Mo ± 0.04%; Cr ± 0.08%.

**Table 2:** Analysis of three experimental steel phase structures.

Code	Type of Precipitated Phase	Type of Crystal System	Lattice Constant/nm
T91LSi	M_23_C_6_	FCC	a_0_ = 1.062~1.064
	Nb(CN)	FCC	a_0_ = 0.440~0.441
	V(CN)	FCC	a_0_ = 0.411~0.412
T91MSi	M_23_C_6_	FCC	a_0_ = 1.062~1.064
	Nb(CN)	FCC	a_0_ = 0.440~0.441
	V(CN)	FCC	a_0_ = 0.411~0.412
T91HSi	M_23_C_6_	FCC	a_0_ = 1.062~1.064
	Nb(CN)	FCC	a_0_ = 0.439~0.440
	V(CN)	FCC	a_0_ = 0.411~0.412

**Table 3:** Amount proportion in M_23_C_6_ phase (at.%).

Code	Cr	Fe	Mo	V	Mn	Ni	C	Σ
T91LSi	50.80	22.15	4.05	1.02	1.00	0.30	20.69	100.00
T91MSi	50.28	22.42	3.97	1.44	0.89	0.31	20.69	100.00
T91HSi	49.95	22.98	3.85	1.26	0.95	0.32	20.69	100.00

**Table 4:** Amount proportion in MX phase (at.%).

Code	V	Nb	Mo	N	C	Σ
T91LSi	37.27	10.88	1.85	34.13	15.87	100.00
T91MSi	34.92	11.70	3.38	34.27	15.73	100.00
T91HSi	35.26	11.25	3.49	33.49	16.51	100.00

**Table 5:** Average oxide film thickness (μm) at different corrosion times static LBE.

Code	50 h	100 h	200 h	500 h	1000 h
T91LSi	5.5	8.6	14	30	41
T91MSi	2.5	6.1	10	18	22
T91HSi	1.3	4.9	8	13	15

## Data Availability

Not applicable.
